# Effects of exercise on executive function in individuals with drug addiction: a systematic review and three-level meta-analysis

**DOI:** 10.3389/fspor.2025.1646327

**Published:** 2025-11-13

**Authors:** Jiawei Chen, Xuan Wang, Xiaofei Zhang, Wenwu Xiao, Anastasiia Kabachkova, Yanchao Tan

**Affiliations:** 1 School of Physical Education and Sport Training, Shanghai University of Sport, Shanghai, China; 2 Faculty of Physical Education, National Research Tomsk State University, Tomsk, Russia; 3 College of Sports Science, Shenyang Normal University, Shenyang City, Liaoning Province, China; 4 Department of Rehabilitation, Chongqing University Three Gorges Hospital, Chongqing, China; 5 Department of Rehabilitation Medicine, Renhe Hospital Affiliated to China Three Gorges University, Yichang, China

**Keywords:** drug addiction, exercise, executive function, meta-analysis, systematic review

## Abstract

**Objective:**

This systematic review and three-level meta-analysis sought to examine the effectiveness of exercise-based interventions on EF among individuals with drug addiction and to determine the role of key moderating factors in this association.

**Methods:**

A systematic database exploration across Embase, Scopus, PubMed, and Web of Science databases was conducted up to April 30, 2025. Eligible studies were randomized controlled trials (RCTs), and their methodological quality was examined utilizing the cochrane risk of bias tool (RoB 2.0). A three-level meta-analysis applying random-effects models was implemented through R software to synthesize findings from RCTs exploring the effects of exercise on EF in individuals with drug addiction.

**Results:**

Eleven RCTs encompassing 906 adult participants with drug addiction were included. Four of the studies were rated as high risk. The findings revealed significant improvements in overall EF [Hedges' g [g] = 0.40; 95% confidence interval [CI] = 0.28, 0.53], as well as in specific EF subdomains: inhibitory control (*g* = 0.25; 95% CI = 0.09, 0.42), cognitive flexibility (*g* = 0.58; 95% CI = 0.27, 0.88), and working memory (*g* = 0.57; 95% CI = 0.34, 0.79). Subgroup analyses identified that aerobic exercise (*g* = 0.38; 95% CI = 0.23, 0.53) and aerobic exercise combined with attentional bias training (*g* = 0.59; 95% CI = 0.33, 0.85) markedly improved EF. Notable improvements of EF were also associated with moderate to vigorous physical activity (*g* = 0.43; 95% CI = 0.30, 0.56), a frequency of 5 times per week (*g* = 0.42; 95% CI = 0.23, 0.60), sessions lasting ≥40 min (*g* = 0.45; 95% CI = 0.30, 0.59), and 12-week interventions (*g* = 0.48; 95% CI = 0.33, 0.64).

**Conclusion:**

Exercise appears to be an efficacious method for improving overall EF and its constituent subdomains among individuals with drug addiction. The effects of exercise on EF are modulated by the specific EF subdomains targeted.

**Systematic Review Registration:**

https://www.crd.york.ac.uk/PROSPERO/view/CRD420251012748, identifier CRD420251012748.

## 1 Introduction

According to statistics provided by the World Health Organization, the 2023 World Drug Report released by the United Nations Office on Drugs and Crime (1) reveals that approximately 296 million individuals globally have engaged in drug use (accounting for 5.8% of those aged 15–64), reflecting a 23% rise compared to figures reported a decade earlier. Drug dependence poses a profound threat to personal physical well-being, frequently resulting in severe medical conditions such as cardiovascular complications, immune system impairment, and organ dysfunction. In addition, it exerts devastating consequences on family units, often resulting in fractured familial relationships and heightened financial burdens (2).

Current mainstream treatment for drug addiction primarily relies on pharmacotherapy and psychosocial interventions. Pharmacological approaches typically involve receptor agonist substitution therapy (e.g., methadone for opioid addiction) or medications targeting withdrawal symptom management (3). However, significant limitations persist: (1) the risk of dependence on substitution medications (e.g., methadone overdose leading to new addiction) (4); (2) limited efficacy of substitution therapies for certain addictions, such as methamphetamine addiction (5); (3) the failure of some agents (e.g., Modafinil) to demonstrably reduce cravings or improve treatment outcomes (6); and (4) the neglect of comorbid psychiatric conditions. Psychosocial interventions, encompassing cognitive-behavioral therapy, group therapy, and various other modalities (7), face challenges including difficulties in achieving sustained treatment goals, variable intervention efficacy, and limited resource accessibility. Exercise interventions emerge as a promising adjunctive strategy to address these shortcomings. Evidence indicates that short-term moderate-intensity aerobic exercise improves inhibitory control and reduces craving in individuals with drug addiction (8). The mood-regulating properties of exercise and its capacity to ameliorate psychosocial comorbidities address key limitations of pharmacotherapy, while its non-pharmacological nature mitigates the risk of addiction substitution. Furthermore, sustained exercise interventions are more likely to promote long-term cognitive improvement and enhance quality of life (9). Although current research is constrained by limited sample sizes and pattern in exercise protocols, the potential of exercise as a cost-effective and safe adjunctive treatment in addiction management warrants further rigorous investigation and validation.

Executive function (EF) plays a critical role in drug withdrawal and recovery. Strong EF abilities facilitate resistance to drug cravings, adherence to rehabilitation plans, and improved treatment efficacy. Whereas EF impairments may contribute to relapse and hinder recovery progress. In recent years, exercise has emerged as a non-pharmacological intervention for enhancing EF in individuals with drug addiction. Accumulating evidence indicates that exercise exerts positive effects on multiple EF subdomains by promoting the secretion of brain-derived neurotrophic factor (BDNF), enhancing neuroplasticity, and improving cerebral blood flow (10). Regarding inhibitory control, aerobic exercise enhances prefrontal cortex (PFC) function and reduces reactivity to drug-related cues. For instance, Zhao et al. (11) demonstrated that 12-week dance or cycling interventions significantly improved electrophysiological markers of inhibitory control in female methamphetamine users. For cognitive flexibility, exercise may facilitate PFC plasticity by modulating BDNF and glutamate systems (12). Furthermore, exercise shows promise for ameliorating impaired working memory in individuals with drug addiction, potentially through BDNF-mediated restoration of synaptic plasticity in the PFC and hippocampus (13). However, current findings present several limitations. First, at the individual level, the efficacy of exercise is influenced by age, gender, substance type (e.g., cocaine, methamphetamine), and duration of addiction, leading to variable responses across individuals and precluding the establishment of universally applicable protocols. Second, substantial heterogeneity exists in intervention protocols regarding exercise type, intensity, and duration, limiting comparability across studies. Finally, most existing research suffers from small sample sizes, a paucity of large-scale randomized controlled trials, and assessment limited to specific EF subdomains. Consequently, the evidence supporting exercise-induced EF improvements in this population remains insufficiently compelling. Current research lacks a meta-analysis directly focusing on the effects of exercise on the executive functions of drug addicts. A meta-analysis (14) has shown that exercise significantly improves negative mood and cognitive function indicators in morphine addicts. However, the impact on executive function subdomains is not explained. And the study population is limited to the individuals with morphine addiction. Moreover, it is still necessary to explore the key moderating factors that may affect the relationship between exercise and executive function.

This study employs a three-level meta-analysis, a method offering distinct advantages over conventional approaches. Traditional meta-analyses assume effect sizes (ES) are mutually independent, typically extracting only one ES per study (15). However, this practice neglects potentially valuable information when multiple ES are reported within a single study. Conversely, the three-level meta-analysis model accounts for both within-study and between-study variability, integrating multilevel data elements such as participant characteristics, experimental designs, and ES metrics into the analytical framework. This enables a more nuanced and accurate estimation of overall effects (16). Given the diversity in EF measurement methods and the abundance of effect sizes reported in the included studies, we applied a three-level meta-analysis to comprehensively synthesize the effects of various exercise intervention protocols on EF changes in individuals with drug addiction. Through rigorous study screening and detailed data extraction, we aimed to precisely control for inter-study confounding factors, fully leverage the potential information within the data, and thoroughly investigate the relationship between exercise characteristics (type, intensity, frequency, duration) and EF improvement. Furthermore, we explored key moderating factors influencing the exercise-EF relationship, such as study design characteristics and sample attributes. The findings are expected to provide a significant theoretical foundation and practical guidance for drug addiction rehabilitation. Clinically, identifying the optimal exercise intervention protocol for enhancing EF in individuals with drug addiction will assist rehabilitation facilities and healthcare professionals in developing more scientific and personalized treatment plans of individuals with drug addiction. This approach holds promise for improving treatment efficacy, reducing relapse rates, and facilitating successful reintegration into daily life. Within the research domain, the results of this three-level meta-analysis will establish a valuable reference framework for future studies, advancing research on exercise interventions in drug addiction rehabilitation and expanding our understanding of effective treatment methodologies.

## 2 Methods

### 2.1 Design and eligibility criteria

This systematic review protocol received registration through the International Prospective Register of Systematic Reviews (PROSPERO registration number: CRD420251012748). The meta-analysis followed the Preferred Reporting Items for Systematic Reviews and Meta-Analyses (PRISMA) guidelines.

The eligibility of studies was determined based on the population, intervention, comparator, outcomes, and study type (PICOS) framework (17), with the following inclusion criteria:

Participants: Individuals diagnosed with drug addiction or substance use disorder by means of validated diagnostic instruments [e.g., diverse editions of the Diagnostic and Statistical Manual of Mental Disorders (DSM)].

Interventions: Studies in which exercise training constituted the primary intervention, encompassing aerobic training, mind-body modalities, or exercise in conjunction with other non-pharmacological treatments.

Comparator: Studies employing non-exercise-based control conditions (e.g., health education sessions, passive rest, or standard treatment routines) as comparators. Outcomes: Studies that included composite EF indicators or reported at least one specific EF-related outcome.

Only RCTs disseminated in peer-reviewed English-language journals were considered.

Exclusion criteria comprised the following: (i) investigations utilizing animal subjects, conference proceedings, book sections, review articles, or unpublished manuscripts; (ii) studies for which ES data could not be retrieved or calculated, even after author correspondence; (iii) trials incorporating exercise within the control group; (iv) research combining exercise with alternative neuromodulation approaches (e.g., transcranial magnetic stimulation); and (v) studies composed in languages other than English.

### 2.2 Literature search

Four English-language databases (Embase, Scopus, PubMed, and Web of Science) were systematically searched from their inception until April 1, 2025, with a subsequent update performed on April 30, 2025. The search protocol incorporated a combination of both keyword terms and Medical Subject Headings, including: Exercise, physical activity, drug addiction, cognition, executive function, and randomized controlled trial. A comprehensive outline of the search procedures is presented in Supplementary File S1. Following the database screening, the titles and abstracts of the retrieved records were independently assessed by two researchers in accordance with the PICOS framework (17). Subsequently, full-text evaluations of the selected articles were conducted on an individual basis. In cases where discrepancies arose between the two reviewers concerning the eligibility of certain studies, a third investigator was engaged to facilitate discussion until a resolution was achieved.

### 2.3 Data extraction and coding

The data extraction process was conducted in alignment with the guidelines outlined in the Cochrane Collaboration Handbook (18). Pertinent data were procured independently by two researchers. The collected variables encompassed the following: reference information (first author), year of publication, sample size (N), average age, sex distribution, diagnostic standards, classifications of drug addiction, intervention groups (experimental and control groups), specific attributes of the exercise interventions (frequency, intensity, time, type, and duration), as well as EF evaluation measures.

From each eligible study, the means and standard deviations (SDs) pertaining to EF baseline and outcome variables in both experimental and control groups were procured and subsequently transformed into mean improvement scores and corresponding standard deviations before and after the intervention. The following computational formula was employed for the estimations (18): mean $\Delta \mathrm{Mean}=\mathrm{Mea}n_{\mathrm{post}\;}-\mathrm{Mea}n_{\mathrm{pre}}$; standard deviation $\Delta SD=\sqrt{SD_{\mathrm{post}}^{2}+SD_{\mathrm{pre}}^{2}-2*0.5*SD_{\mathrm{pre}}*SD_{\mathrm{post}}}$. In instances where original values were absent or inaccessible, efforts were undertaken to obtain the raw data by contacting either the first author or the corresponding author. Five studies reported multiple outcome indicators corresponding to the identical EF subdomain (e.g., both digit backward span and controlled oral word association tests for working memory), all available metrics were incorporated. For publications that included two exercise intervention groups, each was analyzed separately in comparison with the control group. When outcome variables were presented at more than one assessment point (e.g., mid-intervention and post-intervention), ES were computed independently for each respective time point.

The coding framework utilized for the analysis of moderation variables was informed by the methodology outlined in a previous review (19) and tailored according to the specific attributes of the included studies. Drawing upon earlier research, EF subdomains were categorized as inhibitory control, working memory, and cognitive flexibility, which collectively constituted the overall EF construct. Exercise-related features were classified as follows: (i) aerobic exercise, aerobic exercise combined with attentional bias training (AEABT), and Tai Chi; (ii) low vs. moderate-to-high intensity (20); (iii) exercise frequency of three or five times per week; (iv) session time of less than 40 min vs. at least 40 min; (v) exercise duration of 4, 8, or 12 weeks; and (vi) intervention types identified as acute or chronic, depending on duration. Regarding study and sample descriptors: (i) types of addiction drugs were coded as heroin and morphine; (ii) duration of drug use classified as less than 8 years or 8 years and above; (iii) mean age grouped as ≤30 years or >30 years; and (iv) sex of participants defined as female, male, or mixed.

### 2.4 Research quality assessment

The Cochrane risk of bias tool (latest version, RoB 2.0) was used to supplement the risk assessment of included studies. This tool has the unique advantage of assessing the risk of bias or degree of bias of interventions, as it combines two measures to achieve a more comprehensive assessment. The tool categorizes randomized controlled trials (RCTs) as having “low risk,” “some concerns,” or “high risk” of bias (21, 22). Two researchers (J.W. and X.W.) independently assessed the risk of bias for the included studies, and any discrepancies were resolved through discussion with a third researcher (J.C.).

### 2.5 Statistical analysis

Statistical analyses were executed utilizing the Metafor package within R software (version 4.4.1). Each included study reported two or more effect size (ES), with some involving comparisons between two exercise groups and a single control group (23, 24), or assessing EF at diverse time points. To address ES dependencies within individual studies, a three-level meta-analytic model incorporating random effects was implemented. This approach accommodated three hierarchical sources of variance: sampling variance of ES (level 1), within-study ES variance (level 2), and between-study ES variance (level 3) (25). The aggregated ES reflecting exercise effects on overall EF and its specific subdomains was estimated utilizing Hedges' g (g) and 95% confidence intervals (CI), applying the restricted maximum likelihood (REML) method. The statistical significance of the level 2 and level 3 variances was assessed via one-sided likelihood ratio tests (LRTs). A notable variance at any level signified heterogeneity among ES, with *p* < 0.05 denoting statistical significance. Sensitivity analyses were executed to assess the effects of potential outliers and highly influential investigations on the overall ES (26). Following the exclusion of identified outliers, the recalculated pooled ES for overall EF was derived using REML estimation. One-sided LRTs were again applied to determine the significance of variances at levels 2 and 3. Heterogeneity was inferred when statistically significant variance was observed at any level, based on the threshold of *p* < 0.05. A positive Hedges' g was interpreted as indicative of greater beneficial effects of exercise training on EF relative to control conditions. The magnitude of the effect was classified as: (1) small (*g* < 0.20), (2) small to medium (*g* = 0.20–0.49), (3) medium (*g* = 0.50–0.79), and (4) large (*g* ≥ 0.80) (19). Between-study heterogeneity was examined utilizing the *I*^2^ statistic, with values ranging from 1%–49%, 50%–74%, and 75%–100% corresponding to low, moderate, and high heterogeneity, respectively (27). Publication bias was assessed through funnel plot inspection and the multilevel extension of Egger's test (28).

The three-level meta-analysis procedure was executed as follows: (1) the overall impact of exercise training on EF was determined by aggregating all EF-related outcomes; (2) subgroup analyses were performed on categorical moderators, which included EF subdomains (inhibitory control, working memory, cognitive flexibility), and study characteristics such as type of addictive substance, duration of drug use, average participant age, sex, intervention classification, exercise intensity, weekly training frequency, session time, total intervention period, and temporal classification of intervention.

## 3 Results

**Figure 1:**
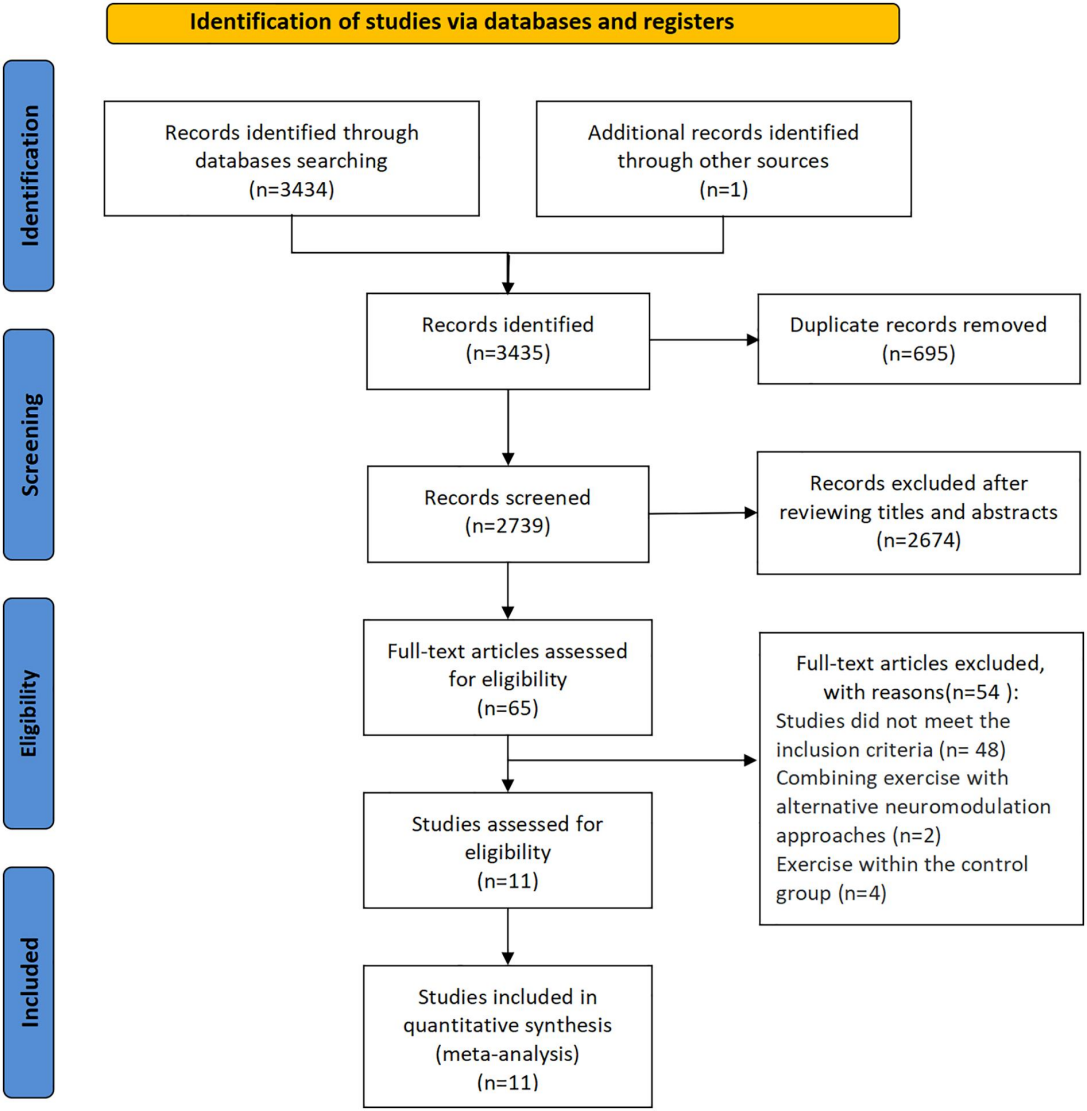
Preferred reporting items for systematic reviews and meta-analyses flow chart of the literature search and selection procedure (29).

Figure 1 presents an overview of the literature retrieval and selection procedure. The initial database search identified 3,434 potentially relevant records, with one additional article identified through alternative sources, totaling 3,435 articles. Following the elimination of 695 duplicate entries, 2,843 records were subjected to title and abstract screening. This screening phase resulted in the identification of 65 full-text articles for further assessment, among which 11 RCTs meeting the predetermined inclusion criteria. Accordingly, all 11 RCTs were incorporated into the final meta-analysis.

### 3.1 Characteristics of included studies

The characteristics of the 11 selected RCTs are depicted in Tables 1, 2. Two-arm and three-arm type randomized controlled trials were included in them. These investigations were published between 2017 and 2024. In total, 906 individuals with drug addiction (aged 20–65 years) were enrolled. The sample sizes across the RCTs varied between 32 and 288 individuals. A cumulative total of 180 female participants was reported. Drug addiction was diagnosed using the DSM Fifth Edition in 8 studies, the Fourth Edition in 2 studies, and the Third Edition in 1 study. In one study, participants were diagnosed with heroin addiction, whereas morphine dependence was reported in the remaining 10 studies. Tai Chi, as a form of mind-body exercise, was utilized in two studies, while aerobic exercise was adopted in 9 studies; among them, 2 employed both aerobic exercise and AEABT as separate intervention arms. Control conditions included quiet sitting, routine care, and health education across the 11 studies. The intervention group training frequencies ranged from 3 to 5 times per week, with session time varying between 20 and 60 min. Low-intensity protocols (Tai Chi) were applied in two RCTs, whereas moderate-to-vigorous intensity regimens were employed in the other 9 trials. The overall duration of exercise programs spanned from a single session to 12 weeks. With respect to EF subdomains, cognitive flexibility was explored in four studies, working memory in six, and inhibitory control in ten.

**Table 1:** Overview of participants' characteristics in the included studies (*k* = 11).

Reference	Year	Sample size (E/C)	Addictive substance	Diagnostic criteria	Duration of drug use	Sex (M/F)	Age (y)
Wang et al. (30)	2017	E: 25	Methamphetamine	DSM-IV	E: 67.39 ± 43.49 (m)	44/6	E: 32.20 ± 6.97
		C: 25			C: 85.13 ± 54.04 (m)		C: 34.76 ± 7.96
Wang et al. (8)	2020	E: 30	Heroin	DSM-V	E: 7.00 ± 6.07 (y)	60/0	E: 32.73 ± 7.15
		C: 30			C: 7.83 ± 5.68 (y)		C: 32.40 ± 7.76
He et al. (31)	2021	E: 16	Methamphetamine	DSM-V	E: 6.69 ± 4.81 (y)	32/0	E: 41.88 ± 13.44
		C: 16			C: 11.25 ± 7.64 (y)		C: 42.81 ± 11.54
Shen et al. (32)	2021	E: 35	Methamphetamine	DSM-V	N	0/72	E: 39.31 ± 10.33
		C: 37					C: 39.37 ± 9.28
Zhu et al. (33)	2022	E: 40	Methamphetamine	DSM-V	E: 8.57 ± 7.14 (y)	77/0	E: 34.61 ± 5.12
		C: 37			C: 8.92 ± 5.31 (y)		C: 34.94 ± 6.58
Liu et al. (34)	2021	E: 142	Methamphetamine	DSM-V	E: 7.32 ± 4.47 (y)	288/0	E: 31.27 ± 5.50
		C: 146			C: 7.28 ± 3.39 (y)		C: 30.92 ± 5.00
Liu et al. (24)	2023	E1: 20	Methamphetamine	DSM-V	E^1^: 8.08 ± 3.10 (y)	66/0	E1: 28.80 ± 3.62
		E2: 23			E^2^: 8.16 ± 3.81 (y)		E2: 28.48 ± 3.30
		C: 23			C: 8.44 ± 3.62 (y)		C: 27.66 ± 3.66
Zhu et al. (35)	2023	E: 30	Methamphetamine	DSM-V	5.35 ± 2.32 (y)	60/0	30.15 ± 3.24
		C: 30					
Zhang et al. (36)	2018	E: 34	Methamphetamine	DSM-V	E: 74.47 ± 46.14 (m)	53/13	33.29 ± 7.74
		C: 32			C: 82.70 ± 59.51 (m)		
Liu et al. (37)	2021	E: 43	Methamphetamine	DSM-III	E: 8.05 ± 3.84 (y)	0/89	E: 29.43 ± 7.91
		C: 46			C: 7.82 ± 3.57 (y)		C: 28.96 ± 8.27
Liu et al. (23)	2024	E1: 24	Methamphetamine	DSM-IV	E1: 7.68 ± 2.97 (y)	60/0	E1: 27.26 ± 3.80
		E2: 23			E2: 7.67 ± 3.53 (y)		E2: 26.63 ± 3.27
		C: 23			C: 7.18 ± 3.16 (y)		C: 27.91 ± 3.13

*k*, number of included studies; y, year, m, month; E, experimental group; E1, first experimental group; E2, second experimental group; C, control group; F, female; M, male; DSM-IV, the Diagnostic and Statistical Manual of Mental Disorders, fourth edition; DSM-Ⅴ, the Diagnostic and Statistical Manual of Mental Disorders, fifth edition; DSM-Ⅲ, the Diagnostic and Statistical Manual of Mental Disorders, third edition; N, Not mentioned.

**Table 2:** Overview of intervention and executive function assessment characteristics in the included studies (*k* = 11).

Reference	Year	Characteristics of exercise	Groups	Executive function assessment
Wang et al. (30)	2017	F: 3 times/week	E: Aerobic exercise (cycling, jogging, or jump rope)	Inhibitory control:Go\No-go test-Accuracy\Reaction Time (Go\No-go-Acc\RT);
		D: 12 weeks	C: Routine care	Go\No-go-N2-Amplitude (Go\No-go-N2-Am)
		T: 30 min		
		I: Moderate-to-vigorous (65%–75% HR max)		
Wang et al. (8)	2020	Acute	E: Acute stationary cycle exercise	Inhibitory control:Go\No-go test-Accuracy (No-go-Acc)
		T: 20 min	C: Sedentary	
		I: Vigorous intensity (70%–80% HR max)		
He et al. (31)	2021	F: 3 times/week	E: Tai Chi	Inhibitory control: Color-Word-Stroop test-Reaction Time (CWS-RT)
		D: 4 weeks	C: Daily routine care	
		T: 45 min		
		I: Low		
Shen et al. (32)	2021	F: 3 times/week	E: Tai Chi	Inhibitory control:Go\No-go test-Accuracy (Go\No-go-Acc);
		D: 12weeks	C: Routine treatment	Working memory:3-Back test-Accuracy\Reaction Time (3-B-Acc\RT)
		T: 40 min		Cognitive reflexibility:Switch test-Accuracy\Reaction Time (Switch-Acc\RT);
		I: Low		
Zhu et al. (33)	2022	F: 5 times/week	E: Aerobic exercise	Inhibitory control:Go\No-go test-Accuracy\Reaction Time (No-go-Acc\RT), Stop-Signal test-Reaction Time (SS-RT)
		D: 4/8/12 weeks	C: Compulsory drug withdrawal procedures without engaging in additional exercise	
		T: 30–36 min		
		I: Moderate		
Liu et al. (34)	2021	F: 5 times/week	E: Aerobic exercise (treadmill)	Inhibitory control: Color-Word-Stroop test-Accuracy\Reaction Time (CWS-Acc\RT)
		D: 8 weeks	C: Health education program	Working Memory: 2-Back task-Accuracy\Reaction Time (2-B-Acc\RT)
		T: 60 min		Cognitive reflexibility: Shift-task-Accuracy\Reaction Time (Shift-Acc\RT)
		I: Moderate-to-vigorous (60%–80% HRmax)		
Liu et al. (24)	2023	F: 5 times/week	E1: Aerobic exercise (treadmill) combined with attentional bias training	Inhibitory control: Color-Word-Stroop test-Accuracy\Reaction Time (CWS-Acc\RT)
		D: 8 weeks	E2: Aerobic exercise (treadmill)	Working Memory: 2-Back task-Accuracy\Reaction Time (2-B-Acc\RT)
		T: 60 min	C: Health education	Cognitive reflexibility: Shift-task-Accuracy\Reaction Time (Shift-Acc\RT)
		I: Moderate-to-vigorous (60%–80% HRmax)		
Zhu et al. (35)	2023	F: 3 times/week	E: Aerobic exercise (cycling, treadmill and lliptical machine)	Inhibitory control: Go\No-go test-Accuracy\Reaction Time (No-go-Acc\RT)
		D: 8 weeks	C: Routine treatment	Working Memory: Sternberg Paradigm task-1\3\5 number-Accuracy\Reaction Time (SP-1\3\5number-Acc\RT)
		T: 40 min		
		I: Moderate-to-vigorous (65%–75% HRmax)		
Zhang et al. (36)	2018	F: 3 times/week	E: Aerobic exercise (cycling, jogging, or jump rope)	Working Memory: 2-Back task-Accuracy\Reaction Time (2-B-Acc\RT)
		D: 12 weeks	C: Routine treatment	
		T: 30 min		
		I: Moderate-to-vigorous (65%–75% HRmax)		
Liu et al. (37)	2021	F: 5 times/week	E: Aerobic exercise (head movements, shoulder movements, chest movements, waist movements, upper limb movements, and lower limb movements.)	Inhibitory control:Color-Word-Stroop test-Reaction Time (CWS-RT)
		D: 12 weeks	C: Routine treatment	
		T: 40 min		
		I: Moderate (55%–69% HRmax)		
Liu et al. (23)	2024	F: 3 times/week	E1: Aerobic exercise (treadmill) combined with attentional bias training	Inhibitory control: Color-Word-Stroop test-Accuracy\Reaction Time (CWS-Acc\RT)
		D: 8 weeks	E2: Aerobic exercise (treadmill)	Working Memory: 2-Back task-Accuracy\Reaction Time (2-B-Acc\RT)
		T: 60 min	C: Health education	Cognitive reflexibility: Shift-task-Accuracy\Reaction Time (Shift-Acc\RT)
		I: Moderate-to-vigorous (60%–85% HRmax)		

*k*, number of included studies; E, experimental group; E1, first experimental group; E2, second experimental group; C, control group; F, frequency; I, intensity; T, time; D, duration; %, percentage; HRmax, maximum heart rate; min, minutes; EF, executive function.

### 3.2 Methodological quality assessment

**Figure 2:**
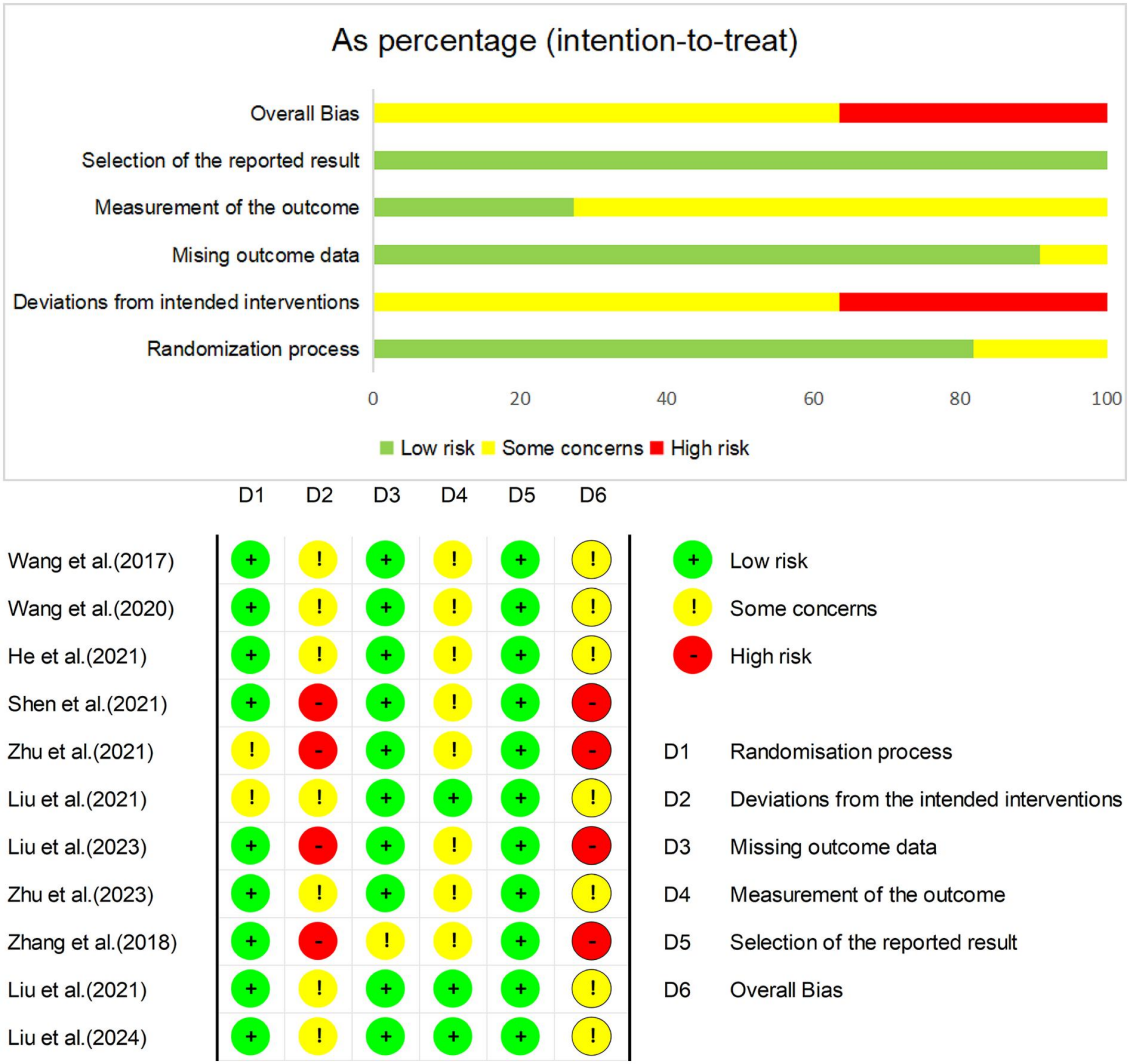
Cochrane RoB 2.0 risk assessment summary.

Based on the results of RoB 2.0, four of the studies were rated as high risk (Figure 2). The domain “Bias due to selection of reported results” had the lowest risk score, while the domain “Bias due to deviations from intended interventions” had the highest risk score. Because the intervention methods between the two groups could not be blinded, this domain had a substantial risk of bias.

### 3.3 Outlier determination and sensitivity analysis

A sensitivity analysis was carried out to examine whether outliers and influential studies affected the overall pooled ES. An individual study, regarded as an outlier, was identified (Supplementary Figures S1A,B). This particular study evaluated working memory based on reaction time within the Sternberg Paradigm task-5 number task following an 8-week intervention (35). Upon exclusion of this outlier, a slight reduction of 0.02 in the pooled ES for exercise on EF was observed, shifting from (*g* = 0.42; 95% CI = 0.28, 0.56; *p* < 0.001) to (*g* = 0.40; 95% CI = 0.28, 0.53; *p* < 0.001). The sensitivity assessment was reiterated, and as presented in Supplementary Table S1, all indices reported I² values of 0, thereby reflecting the stability of the results.

### 3.4 Overall executive function

**Figure 3:**
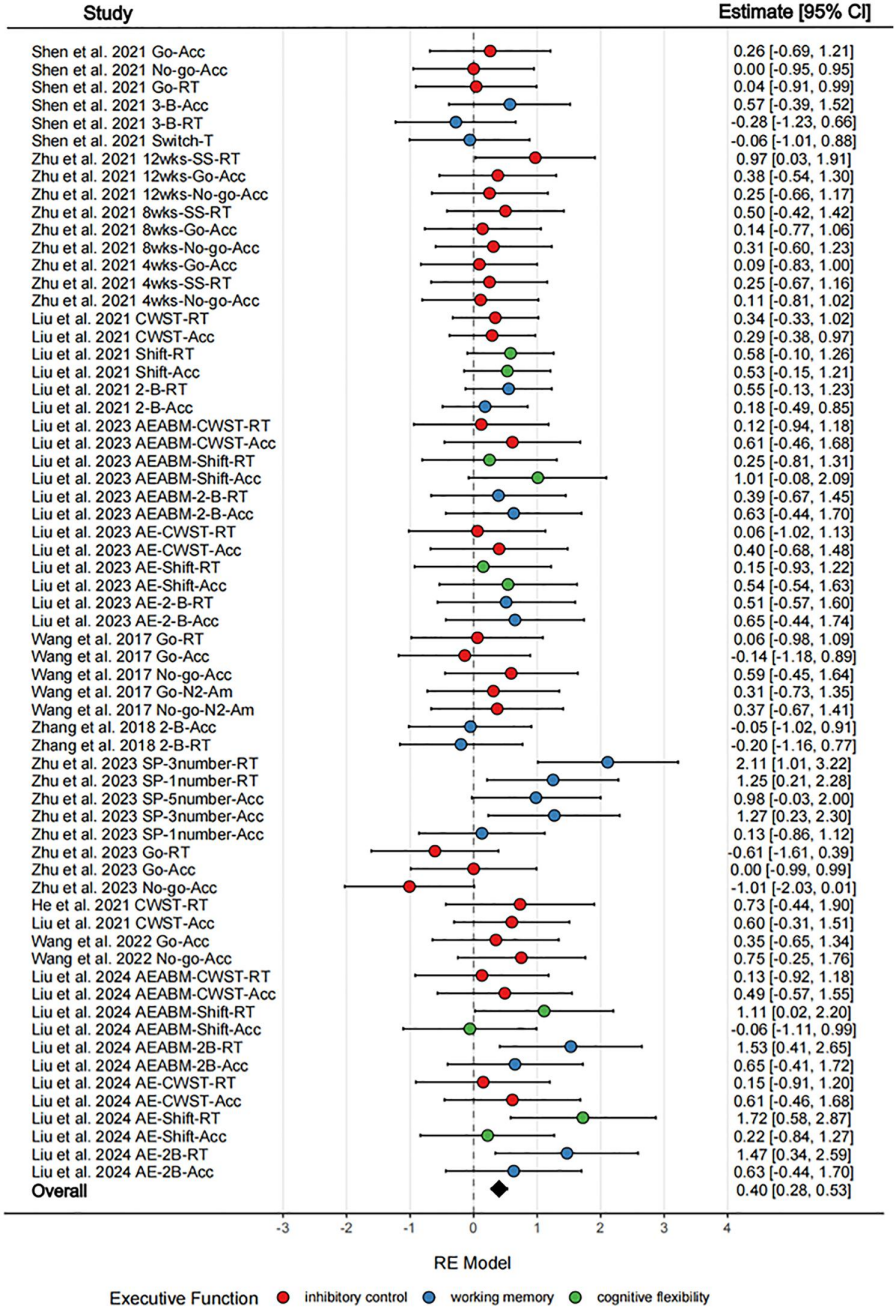
Forest plot of exercise effects on executive function. RE, Model random-effects model; wks, weeks; Go-Acc, Go test-Accuracy; Go-RT, Go test-Reaction Time; No-go-Acc, No-go test-Accuracy; No-go-RT, No-go test-Reaction Time; Go-N2-Am, Go-N2-Amplitude; No-go-N2-Am, No-go-N2-Amplitude; CWS-RT, Color-Word-Stroop test-Reaction Time; CWS-Acc, Color-Word-Stroop test-Accuracy; 2-B-Acc, 2-Back task-Accuracy; 2-B-RT, 2-Back task-Reaction Time; 3-B-Acc, 3-Back test-Accuracy; 3-B-RT, 3-Back test-Reaction Time; Switch-Acc, Switch test-Accuracy; Switch-RT, Switch test-Reaction Time; SS-RT, Stop-Signal test-Reaction Time; 2-B-Acc, 2-Back task-Accuracy; 2-B-RT, 2-Back task-Reaction Time; Shift-Acc, Shift-task-Accuracy; Shift-RT, Shift-task-Reaction Time; SP-1number-Acc, Sternberg Paradigm task-1 number-Accuracy; SP-1number-RT, Sternberg Paradigm task-1 number-Reaction Time; SP-3number-Acc, Sternberg Paradigm task-3 number-Accuracy; SP-3number-RT, Sternberg Paradigm task-3 number-Reaction Time; SP-5number-Acc, Sternberg Paradigm task-5 number-Accuracy; SP-5number-RT, Sternberg Paradigm task-5 number-Reaction Time.

The analysis demonstrated that exercise training (ES: 64, *k* = 11) yielded a statistically significant small-to-moderate ES on overall EF (*g* = 0.40; 95% CI = 0.28–0.53; *p* < 0.001; Figure 3). Results of the LRT indicated that no significant variance was detected within studies (level 2; LRT = 0, *p* > 0.05). But significant variance was detected between studies (level 3; LRT = 16.60, *p* < 0.001), explaining 57.33% of the variance in level 3. The aggregated heterogeneity of the ES was classified as moderate (*I*^2^ = 57.33%).

### 3.5 Moderation effect analysis

#### 3.5.1 Subgroups of executive function

The outcomes of the subgroup analysis are presented in Table 3. Moderation testing revealed statistically significant variations among EF subdomains (*F* = 3.360, *p* = 0.041), indicating that the link between exercise and EF in individuals with drug addiction may be influenced by the specific EF domain assessed. The findings further demonstrated that exercise resulted in a notable improvement in inhibitory control, reflected by a small-to-medium ES (*g* = 0.25; 95% CI = 0.09, 0.42; *p* = 0.004), produced a medium effect on cognitive flexibility (*g* = 0.58; 95% CI = 0.27, 0.88; *p* < 0.001), and was associated with a medium effect on working memory (*g* = 0.57; 95% CI = 0.34, 0.79; *p* < 0.001).

**Table 3:** Moderator analyses of effects of exercise on executive function outcomes.

Subgroup analyses	*k*	Hedges' g (95% CI)	*p*	Test of moderators
Sub-domains				F(2, 61) = 3.360, *p* = 0.041
Inhibitory control	34	0.25 (0.09, 0.42)	0.004	
Cognitive flexibility	10	0.58 (0.27, 0.88)	<0.001	
Working memory	20	0.57 (0.34, 0.79)	<0.001	
Diagnostic tools				F(2, 61) = 0.859, *p* = 0.429
DSM-III	6	0.41 (0.13, 0.70)	0.005	
DSM-IV	17	0.55 (0.28, 0.81)	<0.001	
DSM-V	41	0.35 (0.19, 0.50)	<0.001	
Addictive substance				F(1, 62) = 0.168, *p* = 0.683
Heroin	2	0.55 (−0.20, 1.30)	0.139	
Methamphetamine	62	0.40 (0.26, 0.53)	<0.001	
Mean duration of using drugs (years)				F(1, 56) = 0.198, *p* = 0.658
<8	36	0.46 (0.30, 0.62)	<0.001	
≥8	22	0.40 (0.18, 0.61)	<0.001	
Mean age (years)				F(1, 62) = 3.336, *p* = 0.073
≤30	25	0.57 (0.35, 0.78)	<0.001	
>30	39	0.33 (0.18, 0.47)	<0.001	
Sex				F(2, 61) = 2.367, *p* = 0.102
Female	7	0.16 (−0.20, 0.53)	0.372	
Male	50	0.47 (0.33, 0.61)	<0.001	
Mixed	7	0.12 (−0.27, 0.51)	0.534	
Exercise types				F(2, 61) = 1.981, *p* = 0.147
AEABT	18	0.59 (0.33, 0.85)	<0.001	
Aerobic exercise	39	0.38 (0.23, 0.53)	<0.001	
Tai Chi	7	0.15 (−0.23, 0.52)	0.431	
Intensity				F(1, 62) = 2.036, *p* = 0.159
Low	7	0.15 (−0.23, 0.52)	0.431	
Moderate-to-vigorous	57	0.43 (0.30, 0.56)	<0.001	
Exercise frequency				F(1, 60) = 0.210, *p* = 0.648
3 times/week	26	0.35 (0.11, 0.58)	0.004	
5 times/week	36	0.42 (0.23, 0.60)	<0.001	
Session time				F(1, 62) = 1.525, *p* = 0.220
<40 min	18	0.25 (0.05, 0.51)	0.019	
≥40 min	46	0.45 (0.30, 0.59)	<0.001	
Exercise duration				F(3, 60) = 1.358, *p* = 0.264
Once	2	0.54 (−0.17, 1.27)	0.135	
4 weeks	4	0.25 (−0.25, 0.74)	0.321	
8 weeks	17	0.22 (−0.02, 0.46)	0.073	
12 weeks	41	0.48 (0.33, 0.64)	<0.001	
Temporal classification of intervention				F(1, 62) = 0.168, *p* = 0.683
Single-session	2	0.55 (−0.18, 1.28)	0.139	
Sustained	62	0.40 (0.26, 0.53)	<0.001	

*k*, number of extracted effect sizes, CI = confidence interval; AEABT, Aerobic Exercise Combined with Attentional Bias Training.

#### 3.5.2 Research and sample characteristics

The moderation analysis indicated that the effects of exercise on EF in people with drug addiction were not markedly moderated by diagnostic tools (*F* = 0.859, *p* = 0.429), addictive drug type (*F* = 0.168, *p* = 0.683), duration of drug use (*F* = 0.198, *p* = 0.658), age (*F* = 3.336, *p* = 0.073), or sex (*F* = 2.367, *p* = 0.102), as depicted in Table 3. According to the subgroup findings, the addictive substance variable suggested that exercise did not yield a statistically significant improvement in EF among individuals addicted to heroin (*g* = 0.55; 95% CI = −0.20, 1.30; *p* = 0.139), while a small-to-moderate significant ES was observed for methamphetamine users (*g* = 0.39; 95% CI = 0.24, 0.53; *p* < 0.001). Regarding drug use duration, the results demonstrated that small-to-moderate significant effects of exercise on EF were evident irrespective of how long the substance had been used. With respect to age, moderate significant effects were identified in participants with a mean age ≤30 years, whereas small-to-moderate significant effects were reported among those older than 30 years. In terms of sex, exercise elicited small-to-moderate significant ES in male participants (*g* = 0.47; 95% CI = 0.33, 0.61; *p* < 0.001), but no notable improvements in EF were found for female participants or those in mixed-sex groups.

#### 3.5.3 Exercise characteristics

There were statistically significant moderating variables identified across exercise characteristics (all *p* > 0.05). Within the subgroup analysis, it was found that both AEABT and aerobic exercise were associated with moderate significant effects (*g* = 0.59; 95% CI = 0.33, 0.85; *p* = 0.011) and small-to-moderate significant effects (*g* = 0.38; 95% CI = 0.23, 0.53; *p* < 0.001) on EF, respectively, with AEABT yielding comparatively greater efficacy. Conversely, Tai Chi did not exert a statistically meaningful effect on EF (*g* = 0.15; 95% CI = –0.23, 0.52; *p* = 0.431).

Analysis of exercise intensity suggested that moderate-to-high intensity physical activity was linked to small-to-moderate significant ES on EF (*g* = 0.43; 95% CI = 0.30, 0.56; *p* < 0.001), whereas low-intensity exercise failed to demonstrate SE (*g* = 0.15; 95% CI = –0.23, 0.52; *p* = 0.431).

With respect to frequency, engagement in exercise three and five times per week resulted in small-to-moderate significant ES on EF (*g* = 0.35; 95% CI = 0.11, 0.58; *p* = 0.004) and (*g* = 0.42; 95% CI = 0.23, 0.60; *p* < 0.001), respectively, with superior benefits observed at the higher frequency.

Regarding individual session time, sessions lasting ≥40 min were associated with small-to-moderate significant ES on EF (*g* = 0.45; 95% CI = 0.30, 0.59; *p* < 0.001). When sessions were <40 min, a small-to-moderate ES on EF was also observed (*g* = 0.25; 95% CI = 0.05, 0.51; *p* = 0.019), although the longer duration yielded improved effects.

Regarding exercise duration, interventions conducted over 0–8 weeks exhibited no significant effect on EF, whereas a 12-week duration was correlated with a small-to-moderate significant ES (*g* = 0.48; 95% CI = 0.33, 0.64; *p* < 0.001). In terms of temporal classification of intervention, sustained interventions produced small-to-moderate significant ES on EF (*g* = 0.40; 95% CI = 0.26, 0.53; *p* < 0.001), while single-session interventions failed to reach statistical significance (*g* = 0.55; 95% CI = −0.18, 1.28; *p* = 0.139) (see Table 3 for details).

#### 3.5.5 Publication bias

**Figure 4:**
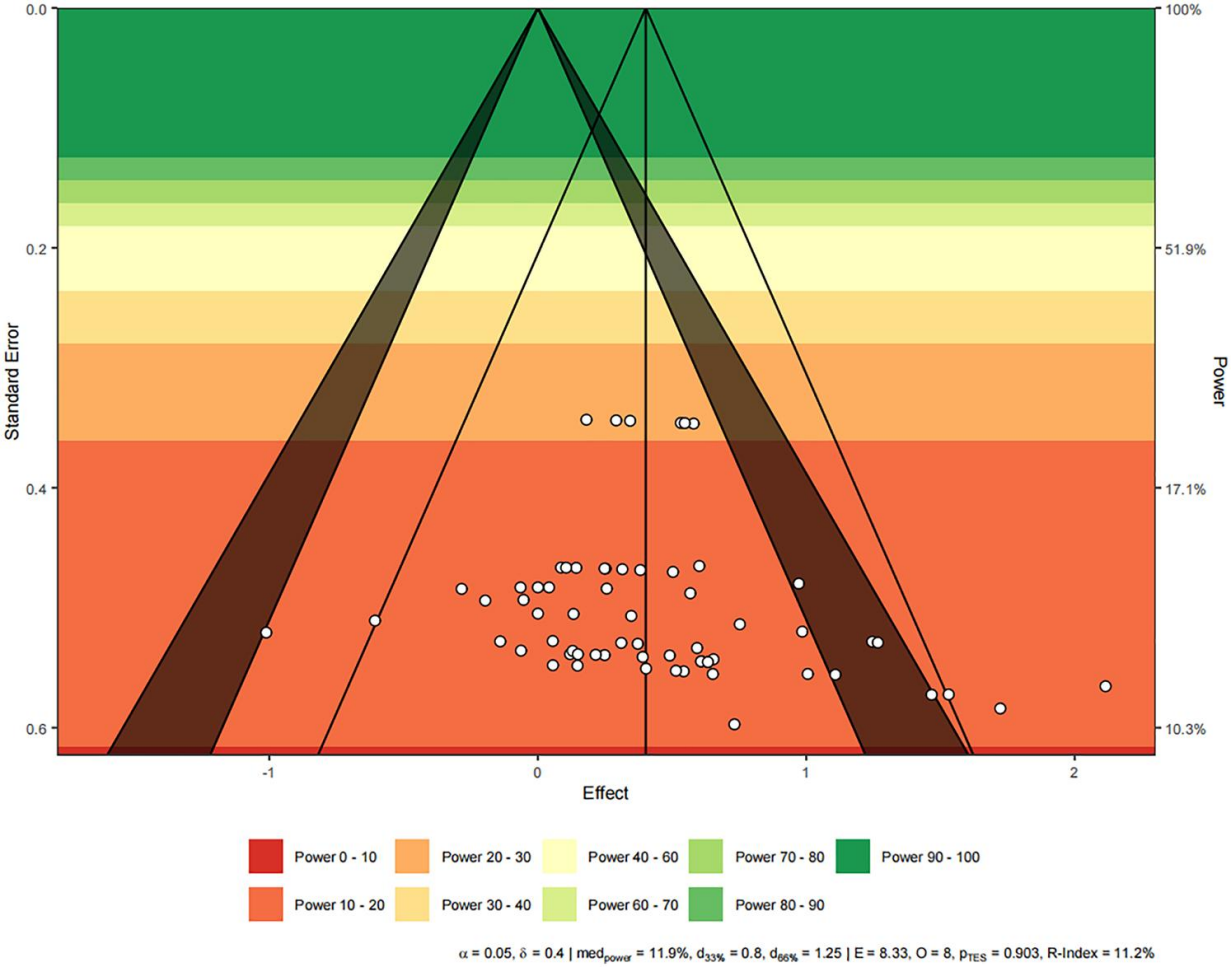
Funnel plot for potential publication bias.

The funnel plot (Figure 4) illustrated a symmetrical arrangement of ES, a pattern further corroborated by the multilevel extension of Egger's test (*t* = 1.356, *p* = 0.180). These findings suggested that publication bias was not detected within the selected studies.

## 4 Discussion

Although substantial empirical support has been established for exercise training as an efficacious adjunct or alternative therapeutic approach for individuals with drug addiction, empirical evidence concerning its effects on EF within this group remains limited. This review constitutes the first known three-level meta-analysis of RCTs exploring the effects of exercise training on EF in individuals with drug addiction. A range of potential moderating variables—including EF subdomains, exercise-related characteristics, and both study-level and sample-level attributes—was systematically evaluated. Eleven RCTs comprising a sum of 809 adult participants with drug addiction were incorporated in the analysis. The findings indicated that exercise exerted a notable overall positive effect on EF in this population. Moreover, EF subdomains were detected to markedly moderate the link between exercise and EF.

### 4.1 Overall executive function

Within this meta-analysis, the advantageous effects of exercise on EF in adults with drug addiction aligns with prior meta-analytical findings involving populations such as those with mild cognitive impairment and dementia (38), as well as children and adolescents irrespective of the presence of EF deficits (39, 40). These outcomes highlight the positive function of exercise in promoting EF among individuals affected by drug addiction.

The observed benefits may be attributed to exercise-induced improvements in neural circuitry underlying executive control (41). Existing research indicates that exercise may reverse drug-induced neural circuit impairments by upregulating dopaminergic signaling, modulating glutamatergic systems, and increasing the expression of neurotrophic factors, thereby contributing to improvements in working memory, inhibitory control, and other domains of EF (12). Moreover, evidence suggests that sustained exercise induces the release of regulatory elements, leading to adaptive modifications in brain structure and function, including strengthened synaptic connectivity, enhanced neurogenesis, and improved structure and functional integrity of the PFC, which collectively promote EF (42). Another potential mechanism might involve morphological and functional alterations within the PFC and anterior cingulate cortex (ACC). These two regions, recognized as pivotal to EF performance, have been reported to exhibit functional impairments and volumetric reductions among individuals with substance dependence (43). It has been documented that physical exercise enhances PFC activation, thereby reinstating inhibitory control, cognitive flexibility, and emotional regulation capacities that have been compromised by addiction, subsequently mitigating impulsive tendencies. Findings presented by Xu et al. (14) emphasize that moderate-to-high intensity aerobic exercise holds particular relevance for such rehabilitative outcomes.

### 4.2 Characteristics of exercise

Five characteristics of exercise training programs—namely, intervention type, intensity, frequency, single-session time, and overall intervention period—have been identified as essential factors in establishing dose-response relationships for exercise-induced improvements in EF among individuals with drug addiction. According to subgroup analyses, both AEABT and aerobic exercise were associated with effective improvements in EF. Nonetheless, Tai Chi should not be classified as ineffective, as multiple studies have validated the beneficial effect of mind-body practices on EF. For instance, a systematic review and meta-analysis suggested that such exercises, including Tai Chi, markedly improved general EF and its subdomains (e.g., working memory and cognitive flexibility) in middle-aged and older adults (29). Moreover, evidence from another investigation demonstrated that Tai Chi practice led to EF improvements accompanied by elevated PFC activity (44). These findings corroborate the advantageous role of Tai Chi in enhancing EF. It is important to acknowledge that only two studies utilizing Tai Chi were incorporated in the present meta-analysis. Consequently, due to this limited dataset, the reliability of subgroup analysis outcomes for this modality remains constrained. Further investigations into Tai Chi-based interventions are warranted to determine their efficacy in improving EF. Concerning exercise intensity, the current study indicates that moderate-to-vigorous intensity regimens contribute to improved EF, aligning with previous meta-analytic findings (19), which demonstrated that moderate-to-high intensity exercise produced significant EF gains in individuals with depression, whereas low-intensity formats yielded no such effect. Similarly, the observed outcomes for exercise frequency (5 times weekly) and session time (≥40 min) are consistent with findings reported in earlier reviews (45). Among the RCTs assessed, the highest frequency applied for EF improvement in individuals with drug addiction was five times per week, a pattern that corresponds to global physical activity guidelines recommending activity on five or more days weekly. Additionally, in terms of temporal intervention design, long-term training interventions have exhibited substantial benefits for individuals with drug addiction—a trend supported by comparable previous research. An expanding body of empirical evidence indicates that EF is responsive to extended exercise exposure (46, 47). Exercise regimens extending over 12 weeks have yielded significant EF improvements, with corroborative findings emerging from analogous studies (48). Current research thus suggests that intervention protocols incorporating moderate-to-high intensity, five weekly sessions, individual durations of ≥0 min, and a total length of 12 weeks offer considerable benefits for EF among drug-addicted populations. These programmatic parameters may serve as a basis for structuring future experimental and clinical frameworks.

The three-level meta-analysis also explored sample-related characteristics. However, no statistically significant moderators were identified in the relationship between exercise and EF outcomes. The findings demonstrated a notable improvement of EF in individuals with morphine addiction. Nonetheless, conclusions regarding the ineffectiveness of exercise for heroin addicts should not be drawn, as prior studies have substantiated the positive effects of exercise interventions on EF among heroin-dependent populations (12). Additionally, it must be acknowledged that only a single study involving heroin addicts was incorporated into this meta-analysis, thereby rendering the subgroup analysis less robust due to the limited evidence base. Of particular interest, the results indicated that EF was markedly improved in studies that included only male participants. From a neurobiological standpoint, elevated testosterone levels observed in males following physical activity may contribute to attenuated dopaminergic responses to drug stimuli, ultimately reducing drug-seeking behaviors (49). However, assertions that exercise lacks effectiveness in female individuals with drug addiction would be premature, as empirical data suggest that both male and female users benefit from exercise, with females exhibiting heightened sensitivity under concurrent conditions, voluntarily prioritizing running over drug intake (50). Furthermore, it is noteworthy that only two studies focusing exclusively on female drug users were included in this analysis, suggesting that the subgroup results warrant cautious interpretation. Continued investigation is essential to further clarify gender-specific effects of exercise on EF improvements.

### 4.3 Advantages and limitations

This three-level meta-analysis offers numerous methodological advantages. Foremost, a three-level meta-analytic approach was implemented initially to explore the effects of exercise on EF and its specific subcomponents in individuals affected by drug addiction. Additionally, various moderating variables were systematically assessed, and preliminary exercise dosage parameters with potential efficacy for improving EF in this population were identified. Nonetheless, a number of limitations merit consideration. First, wide variety of tasks may not be equivalent across subdomains. Second, in an effort to uphold methodological rigor and improve the reliability of findings, only peer-reviewed English sources were considered, which precludes any assessment of the file drawer effect. Third, most of the 11 RCTs incorporated in the analysis lacked follow-up evaluations. Consequently, post-intervention durability of exercise-related EF improvements could not be examined. Future investigations should address the duration of such benefits. Forth, all included studies come from China, which increased the homogeneity between studies to a certain extent, but also severely limited the cross-cultural universality of the findings. China's specific drug treatment policies and socio-cultural context may have influenced the effectiveness of interventions, so caution is needed when generalizing results to other countries and regions. Fifth, the included studies focused on methamphetamine and heroin addicts, and the conclusions of this analysis may not apply to other types of drug addiction (such as cocaine or cannabis addiction) given the different mechanisms of impairment of executive function neural circuits by different drugs. Moreover, the female sample in the included studies was severely underrepresented, preventing us from exploring potential gender differences in the effectiveness of exercise interventions. Future research will be suggested to include a wider range of drug types and a sufficient sample of women to validate and expand the findings of this study and provide evidence for the development of personalized rehabilitation programs. Lastly, the RCTs analyzed did not provide explicit reporting regarding the severity of drug addiction among participants. Given that addiction severity may serve as a meaningful moderating variable, subsequent RCTs are encouraged to offer precise documentation of symptom severity.

## 5 Conclusion

This three-level meta-analysis reveals that exercise exhibits promising capabilities to improve overall EF in individuals with drug addiction, offering measurable therapeutic value in the context of withdrawal rehabilitation. Aerobic exercise performed at moderate to high intensity, with sessions lasting ≥40 min, five times weekly across a 12-week intervention period, appears to yield the greatest benefits for EF, particularly in those dependent on morphine. Although these findings are encouraging and may inform clinical practice in substance rehabilitation settings, existing literature on this complex and sensitive issue remains scarce. Further investigations are necessary to elucidate the particular effects of exercise on EF and its subcomponents in individuals affected by drug addiction.

## Data Availability

The original contributions presented in the study are included in the article/Supplementary Material, further inquiries can be directed to the corresponding author/s.
