# Supplementary figures and images for: Effects of exercise on executive function in individuals with drug addiction: a systematic review and three-level meta-analysis
Source: Front Sports Act Living. 2025 Nov 13;7:1646327. doi: 10.3389/fspor.2025.1646327 (PMC12657471; doi:10.3389/fspor.2025.1646327)

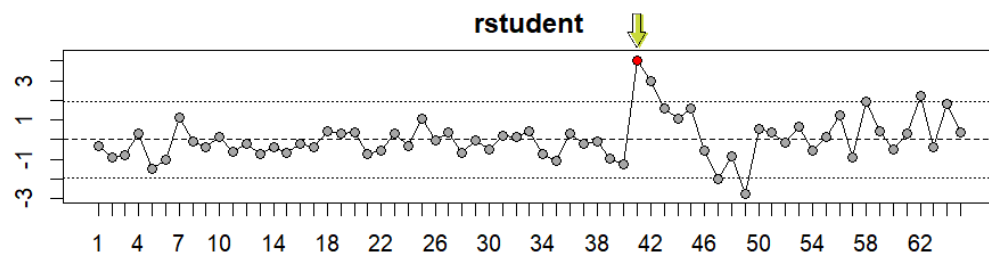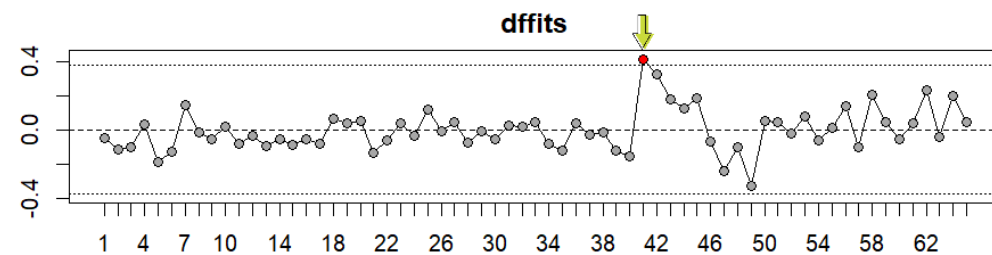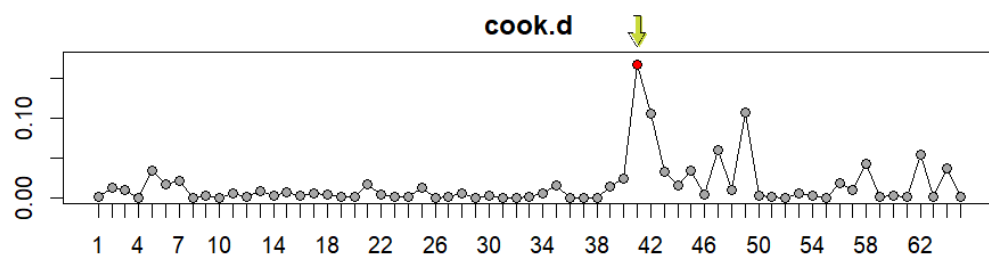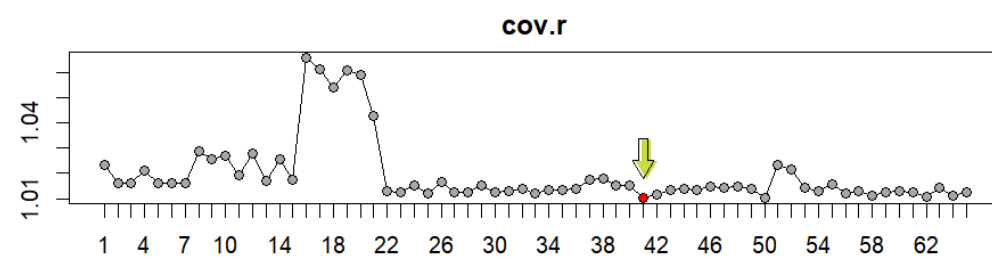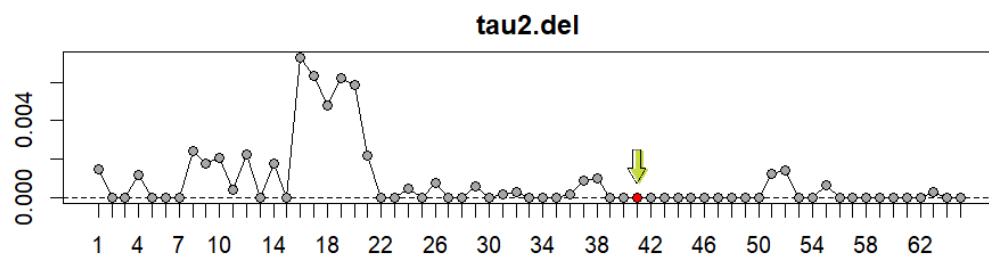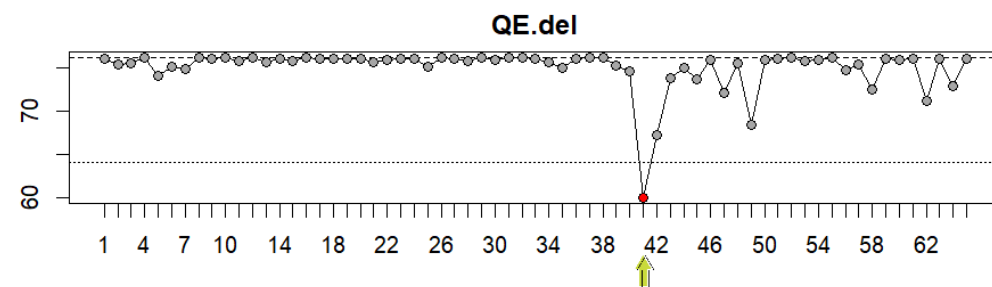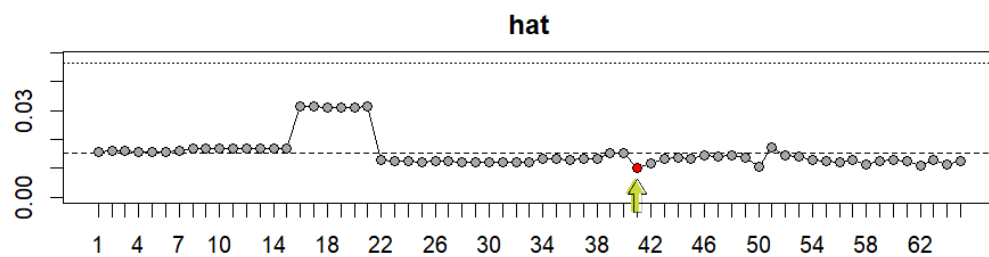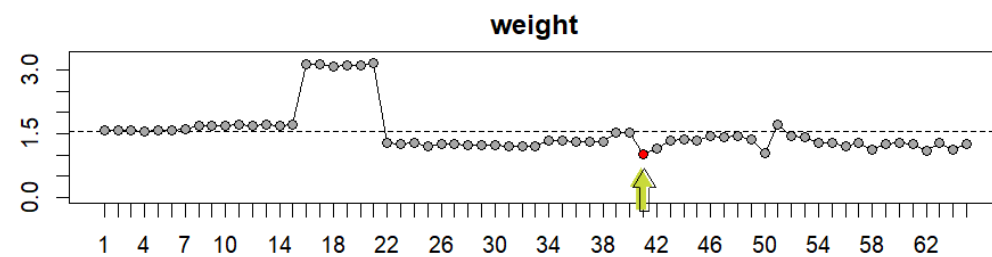

Supplement: Supplementary file 1 [file Datasheet1.pdf]

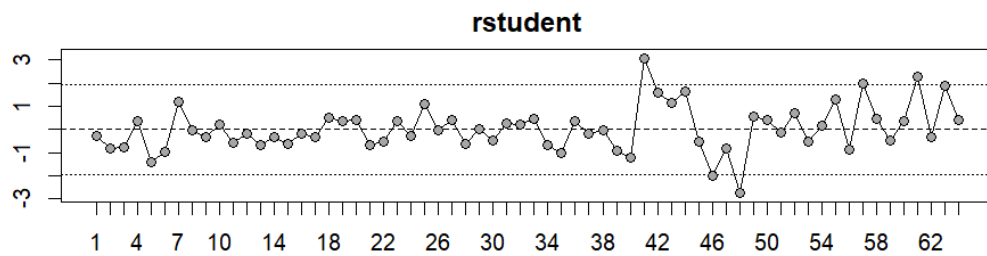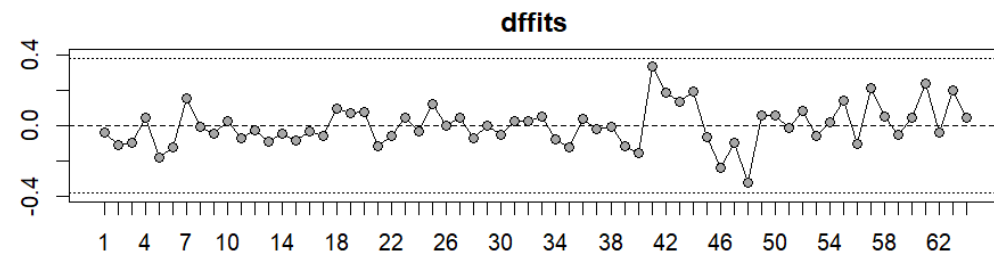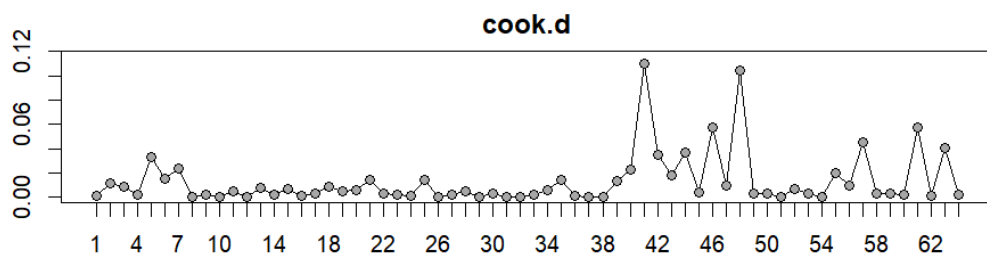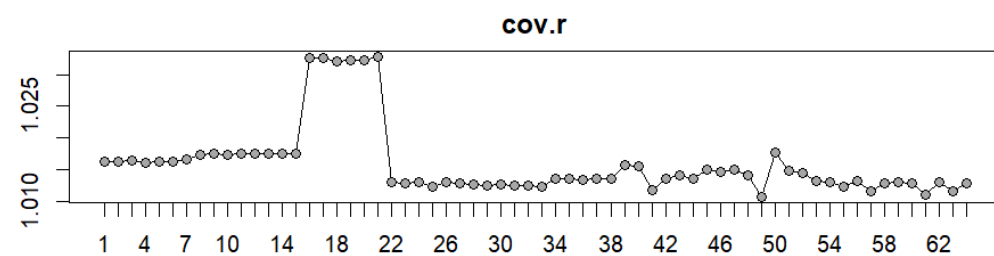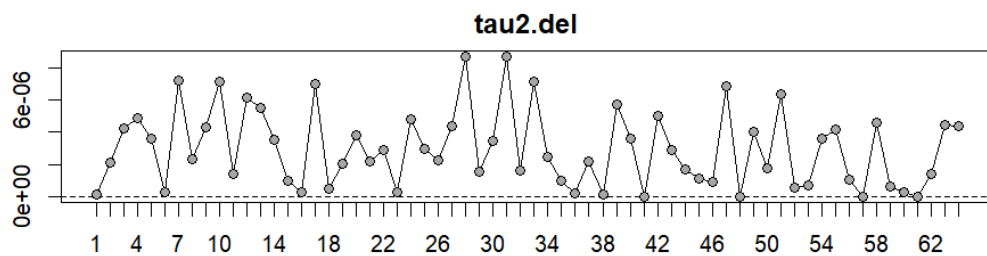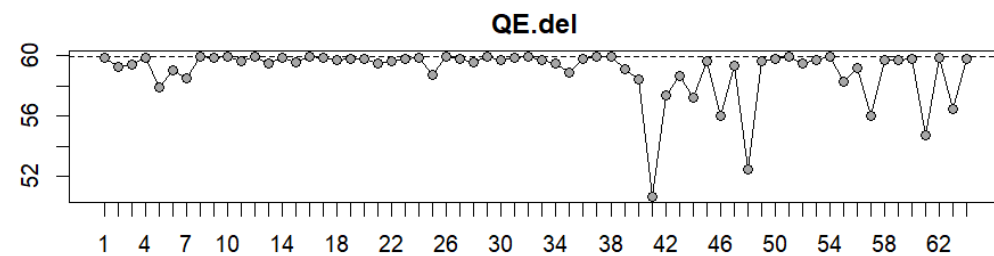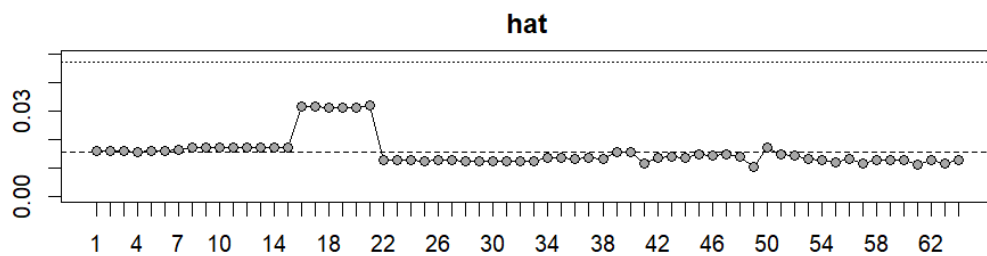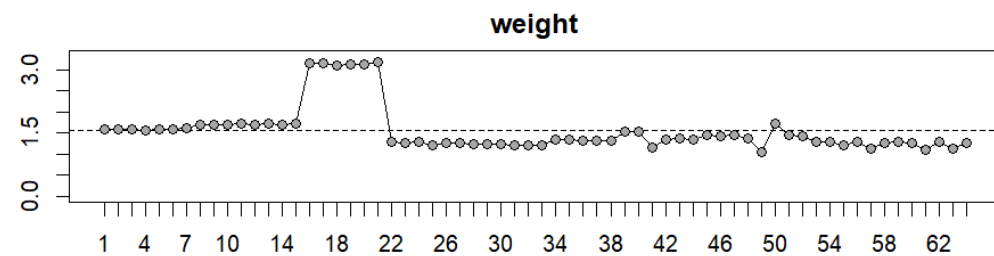

Supplement: Supplementary file 2 [file Datasheet2.pdf]
